# Lichenoid lesions of the upper lip: A retrospective study of 24 cases

**DOI:** 10.4317/medoral.22303

**Published:** 2018-04-24

**Authors:** Nikolaos Katsoulas, Konstantinos Tosios, Alexandra Sklavounou-Andrikopoulou

**Affiliations:** 1 DDS, MSc, MSc in Oral Medicine and Pathology. Department of Oral Medicine and Pathology, School of Dentistry, National and Kapodistrian University of Athens, Athens, Greece; 2 DDS, PhD, Assistant Professor. Department of Oral Medicine and Pathology, School of Dentistry, National and Kapodistrian University of Athens, Athens, Greece; 3 DDS, MSc, PhD, Professor. Department of Oral Medicine and Pathology, School of Dentistry, National and Kapodistrian University of Athens, Athens, Greece

## Abstract

**Background:**

Lichenoid lesions of the upper labial mucosa, without other oral or extraoral manifestations seem to be rare. The clinicopathologic features of 24 such cases are presented and the pertinent literature is reviewed.

**Material and Methods:**

24 Caucasian patients that clinically presented lichenoid features on the upper labial mucosa, with or without lichenoid lesions on the adjacent gingiva, were included in the study. Clinical features were extracted from the patients’ records, while dental plaque/calculus accumulation and composite resin restorations of the adjacent teeth were recorded. Four cases where an incisional biopsy was performed were further evaluated.

**Results:**

There were 8 males and 16 females, with a mean age of 62.7 years. 64.2% were under hypertensive therapy. In 13 cases gingival involvement was noticed, 16 cases exhibited calculus deposition, while 6 cases presented with composite resin fillings of the adjacent teeth. In 4 cases an incisional biopsy was performed showing features of lichenoid reaction. In 37.5% significant improvement was observed after topical treatment with corticosteroids and antimicrobial agents.

**Conclusions:**

Lichenoid lesions of the upper lip may represent a distinct variety of oral lichenoid lesions, but as the number of cases reported so far is too small for definite conclusions on pathogenesis and management to be made, a long-term follow-up is mandatory.

** Key words:**Lichenoid lesions, upper lip, oral lichen planus, lichenoid reactions.

## Introduction

Lichenoid lesions of the oral mucosa encompass a spectrum of oral pathoses, ranging from lichen planus, to graft-versus-host disease and lichenoid reactions due to systemically administered drugs or dental restorations. In particular, lichen planus is an immune-mediated mucocutaneous disease that commonly manifests exclusively to the oral mucosa, without skin or other mucosal manifestations (1). It affects 0.5 to 2% of the population, mostly women, and it typically presents with white striae, usually accompanied by erythema, erosions, white plaques or atrophy that have a characteristic bilateral distribution. The buccal mucosa and tongue are the most frequently involved sites (1,2).

Oral lichenoid lesions have similar clinicopathologic features with lichen planus and represent a form of hypersensitivity reaction triggered mainly by dental restorative materials or drugs (2). In contrast to lichen planus, oral lichenoid lesions may be unilateral and develop adjacent to the offending dental material or following the administration of certain drugs (1).

Lichen planus and lichenoid reactions are commonly seen in the daily practice of Oral Medicine, but lichenoid lesions confined to the labial mucosa, without any other oral or extraoral manifestations seem to be rare (3,4). Historically, cases of lichenoid lesions confined particularly to the lower vermilion border and labial mucosa, not associated with drugs, dental materials, or dental plaque, were mainly described by dermatologists (3-6). Backman and Jontell (7) reported 25 cases of lichenoid lesions of the upper lip with accompanying gingival involvement and suggested that they may represent a microbial-induced lichenoid inflammation.

Although these lesions have distinctive clinical features, their pathogenesis and management remain obscure. Therefore, the clinicopathologic features of 24 cases of lichenoid lesions of the upper labial mucosa are presented and the pertinent literature is reviewed.

## Material and Methods

This retrospective study included patients presenting with lichenoid lesions of the upper lip to the Stomatology Clinic during the years 2010-2014. Lichenoid lesions were defined as white striations associated with erythema/erosions on the upper labial mucosa that might coexist with similar lesions on the adjacent labial gingival mucosa (7). Patients with lichenoid lesions on other oral mucosal sites or the skin were excluded from the study. The age and sex of the patients, time between presentation and diagnosis, medical and dental history, and clinical signs and symptoms were retrieved from the patients’ records. Dental plaque/calculus accumulation and composite resin restorations of the adjacent teeth were recorded during clinical examination. Incisional biopsies were performed in four cases and 5μm thick, formalin-fixed and paraffin-embedded tissue sections were stained with hematoxylin and eosin, PAS, and Gram stains. Polarized light examination for the identification of foreign bodies was also performed. Specimens were, also, stained for CD68 protein (monoclonal mouse anti-human PG-M1 clone, 1:100 dilution, Dako® technologies) with a standard avidin-biotin-peroxidase immunohistochemical technique (Vectastain Elite® ABC kit-Vector laboratories).

In order to be eligible for diagnosis and treatment in our institution, each patient has to sign an informed consent form that also allows the use of any available material (patient’s archive, clinical images etc) for scientific research. Our work was approved by the institutional Ethics and Research Committee (approval no. #251A).

## Results

Twenty-four Caucasian patients examined during the 5 years period met the inclusion criteria of our study ( Table 1). There were 8 male (33.3%) and 16 female patients (66.7%), and the mean age was 62.7 years (±14.2 years, age range: 22-82 years). The mean time between presentation of the lesion and diagnosis was 5.56 months, but in many cases the patients could not recall when they first noticed the clinical signs. Regarding medical history, 64.2% of patients were under therapy for hypertension or other cardiovascular disease with beta-blockers (47%), calcium channels blockers (38%) or angiotensin converting enzyme inhibitors (15%).

**Table 1:**
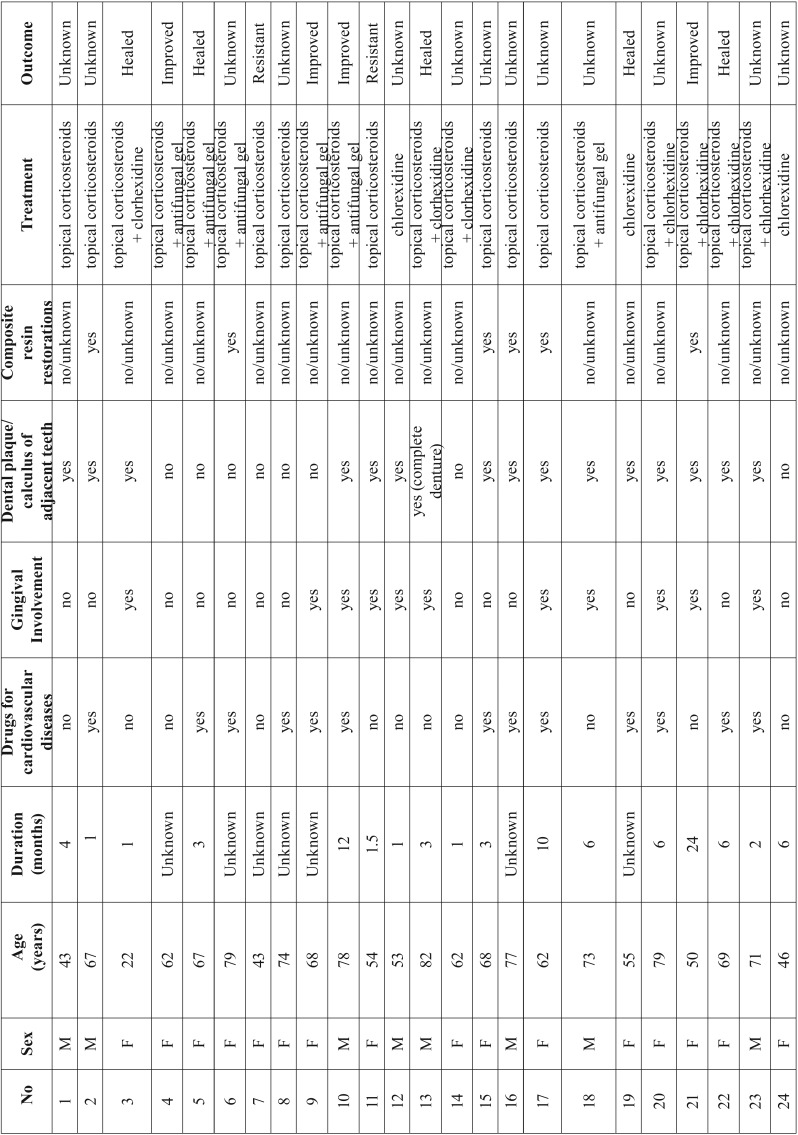
Main clinicopathologic features of the 24 cases.

**Figure 1:**
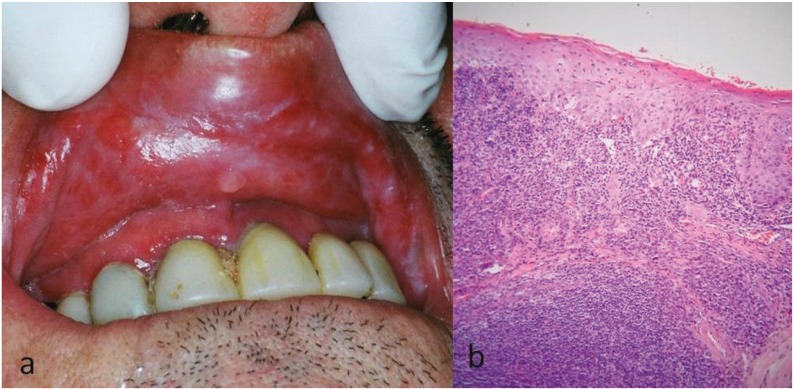
Patient #12. (a) Clinical picture. White striae and erythema of the upper labial mucosa, accompanied by gingival erythema. Dental plaque accumulation on the upper incisors is noticed. (b) Microscopic picture. Hydropic degeneration of basal cells, and subepithelial band-like and focally perivascular lymphohistiocyte inflammatory infiltrate (hematoxylin and eosin stain, original magnification x100).

Clinically, all patients exhibited white striae, erythema and/or erosions on the upper labial mucosa, while in 13 cases (54.2%) erythema of the upper labial gingival mucosa, resembling desquamative gingivitis was also present (Fig. 1a). Most patients reported pain and discomfort to the affected area that in some of them were intense and interfered with mastication and oral hygiene.

In 66.7% of cases (n=16) dental plaque and calculus deposition on the upper incisors and canines was noted. Interestingly, one patient with a complete upper denture showed lichenoid lesions in contact with calculus of the anterior facial aspect of the denture. Concerning dental restorations, in 25% (n=6) of the patients composite resin fillings were noticed in the adjacent teeth that were mostly old and of poor quality.

In four cases, the aforementioned clinical features were not pathognomonic; as a result, an incisional biopsy was performed in order to confirm the diagnosis, under local infiltration anesthesia. Microscopically, features typical of oral lichen planus/lichenoid reaction were seen, i.e. hydropic degeneration of basal cell layer, Civatte bodies, and lymphohistiocytic inflammatory infiltrate with a subepithelial band-like or, focally, perivascular distribution (Fig. 1b). PAS and Gram stains did not reveal the presence of superficial or deep fungal or bacterial colonies, respectively; polarized light did not identify foreign bodies; focally intense infiltrate of the lamina propria by numerous CD68(+) histiocytes that did not form granulomas was noticed.

**Figure 2:**
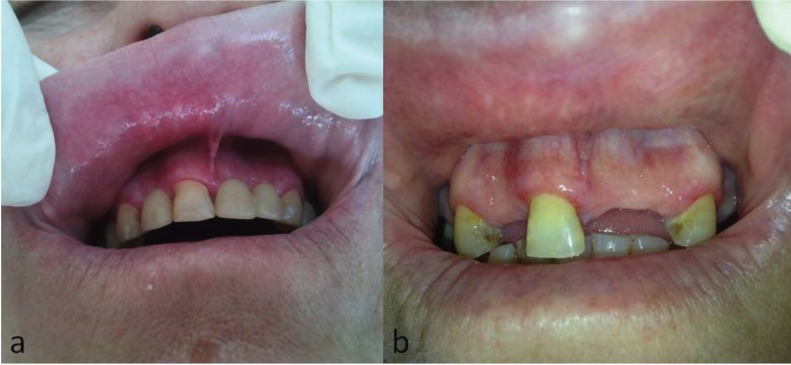
Patient #21. (a) Striation and erythema of the upper labial mucosa and upper facial gingiva. (b) Same patient, complete resolution of lesions after topical application of corticosteroid ointment and chlorhexidine gel. Teeth extractions were performed due to periodontitis after lesions had healed.

The patients were diagnosed and managed by different staff members of our Department, with different medications. In 8 cases topical application of corticosteroids, either as ointment or as oral mouthwash was prescribed. In the remaining cases, the combination of topical corticosteroids with a chlorhexidine gel or antifungal gel, and in 3 cases chlorhexidine gel as monotherapy, were given. The outcome of the above therapeutic interventions varied from total resolution (n=9, 37.5%) to significant clinical improvement (n=4, 16.7%); lack of improvement was noticed in 2 cases (n=2, 8.4%) (Fig. 2). Eleven patients were on a 12 months follow-up and no new lesions developed. None of the six patients where replacement of the resin fillings was instructed presented on the follow-up examination.

## Discussion

The patients described herein showed lichenoid lesions on the upper labial mucosa and, in approximately half of the cases, the adjacent gingival mucosa. Lip lesions may represent the first manifestation of oral lichen planus, but upper lip involvement by this disease is seen only in 13% of cases (3). In our patients, no other oral mucosa site was involved and no cutaneous lesions developed during a 12-month follow-up period, while in one case there was complete resolution of the lesion following chlorhexidine monotherapy. Drug-induced lichenoid reactions are indistinguishable from oral lichen planus and are associated with a variety of drugs, in particular non-steroidal anti-inflammatory drugs, anticonvulsants, antibiotics, antiretroviral, antimalarians and anti-hypertensives (2). They may develop within days, weeks, months, even a year since the initiation of the medication (2). In Backman and Jontell’s series, patients with lichenoid lesions of the upper lip were statistically more often under drug treatment, even with more than one drug, compared to those diagnosed with lichen planus. No association was mentioned between drug intake and development of the lesions, save for a possible correlation with drug-induced xerostomia that along with mouth breathing could enhance dental plaque accumulation on dry tooth surfaces (7). Approximately 2/3 of our patients were under medication for cardiovascular disease and none complained for dry mouth, although xerostomia or mouth breathing were not investigated. In 1/3 of our patients, however, a drug induced side effect cannot be hypothesized. As none of our patient had undergone allogenic bone marrow transplantation, lichenoid lesions of graft versus host disease was not considered in the differential diagnosis (2).

Oral lichenoid reactions to composite resins are documented in the literature (8) and resin fillings were present on the facial aspect of the upper incisors in 24 of the 25 cases by Backman and Jontell (7), but in just ¼ of our patients. Although hypersensitivity reaction to formaldehyde-containing resins could be involved in the pathogenesis of those lesions, Blomgren *et al.* (8) hypothesized that disruption of the oral mucosa barrier by a rough surface material or topical action of formaldehyde facilitates colonization and infiltration by *Candida* sp or other microorganisms, initiating and sustaining a lichenoid inflammatory process. They identified *Candida* hyphae in 7 of 12 cases by microbial culture or histopathologic examination and found that replacement of the material with acrylic, porcelain or glass ionomer, in parallel with topical antifungal and corticosteroid therapy resulted in resolution of the lesions. Components of *Candida’s* sp cellular antigens may trigger a delayed hypersensitivity reaction, as has been documented in recurrent vulvovaginal candidiasis that is associated with an IgE-mediated immunologic response to structural elements of the yeast (9). In addition, in a case of isolated labial lichen planus, superficial colonization by *Candida famata* was found and total resolution of the lesion was noticed after treatment with corticosteroids and griseofulvin (10). Backman and Jontell (7) were, also, in favor of a microbial cause for lichenoid lesions of the upper labial mucosa, most probably to components of the dental plaque and calculus accumulated on the facial aspects of the upper incisors, including *Candida* sp. In most cases replacement of resin fillings and/or topical chlorhexidine application had a therapeutic effect. An allergic reaction to zinc, another component used in various dental materials such as adhesives of composite resins, has also been associated with the development of annular atrophic lichen planus of the lip vermillion (11). A common finding in most of our cases was the presence of dental plaque and calculus deposition on the upper incisors and canines, while in one case resolution of the lesions followed the topical application of chlorhexidine. However, microbes, *Candida* hyphae and foreign bodies were not found in the four biopsized cases and Ziehl-Nielsen for mycobacteria was negative, as has been previously reported (12). It is noticed that PAS stain is the gold standard for identifying Candida hyphae (18).

Backman and Jontell (7) suggested that histopathologic examination would not be contributory to the final diagnosis, as lichen planus and lichenoid lesions of the oral mucosa share the shame pathological features (1). In a retrospective analysis of 6 patients exhibiting erythema occasionally accompanied by swelling of the upper labial mucosa, lichenoid and granulomatous inflammation of the lamina propria was seen and the name “lichenoid and granulomatous stomatitis” was proposed, as the oral counterpart of “lichenoid and granulomatous dermatitis” (12). In our cases where an incisional biopsy was performed, a non-diagnostic lichenoid inflammation was seen. Immunohistochemical staining for CD68 showed focally intense infiltration of the lamina propria by numerous CD68(+) histiocytes that is in accordance with the immunophenotype of conventional lichen planus (13), but no granulomas.

Backman and Jontell (7) administered topical chlorhexidine in 80% of patients with lichenoid lesions of the upper lip with significant improvement or total resolution. In three patients to whom corticosteroids with or without topical antifungals were applied the results were variable. Blomgren *et al.* (8) showed that replacement of composite resin by porcelain or acrylic resulted to resolution of the lesions, in contrast to the application of topical corticosteroids and antifungals that provided temporary relief. In other lichenoid lesions of the lower lip resolution was reported after treatment with topical corticosteroids, tacrolimus, retinoids, imiquimod, clarithromycin or chloroquine (3-6,14-17). Treatment modalities in our patients did not follow a certain protocol and varied from topical corticosteroid application to topical antimicrobial agents or combinations, based on the judgment and experience of the clinician involved. Total resolution of the lesions was noticed in one patient only to whom chlorhexidine gel was prescribed as monotherapy; other cases with total or partial healing of the lesions were treated with combination of antimicrobial agents and corticosteroid ointments.

In summary, lichenoid lesions of the upper labial mucosa may represent a distinct variety of oral lichenoid lesions, but as the number of cases reported so far is too small for definite conclusions on pathogenesis and management to be made, a long-term follow-up is mandatory.

## References

[1] van der Waal I (2009). Oral lichen planus and oral lichenoid lesions; a critical appraisal with emphasis on the diagnostic aspects. Med Oral Patol Oral Cir Bucal.

[2] Schlosser BJ (2010). Lichen planus and lichenoid reactions of the oral mucosa. Dermatol Ther.

[3] Itin PH, Schiller P, Gilli L, Buechner SA (1995). Isolated lichen planus of the lip. Br J Dermatol.

[4] Petruzzi M, De Benedittis M, Pastore L, Pannone G, Grassi FR, Serpico R (2007). Isolated lichen planus of the lip. Int J Immunopathol Pharmacol.

[5] Yu TC, Kelly SC, Weinberg JM, Scheinfeld NS (2003). Isolated lichen planus of the lower lip. Cutis.

[6] Cecchi R, Giomi A (2002). Isolated lichen planus of the lip. Australas J Dermatol.

[7] Bäckman K, Jontell M (2007). Microbial-associated oral lichenoid reactions. Oral Dis.

[8] Blomgren J, Axéll T, Sandahl O, Jontell M (1996). Adverse reactions in the oral mucosa associated with anterior composite restorations. J Oral Pathol Med.

[9] Bernstein JA, Seidu L (2015). Chronic vulvovaginal Candida hypersensitivity: An underrecognized and undertreated disorder by allergists. Allergy Rhinol (Providence).

[10] Chiang CT, Chan HL (2002). Superficial mycosis superimposing on isolated lichen planus of the lip: a case report and reviewof the literature. Cutis.

[11] Sugashima Y, Yamamoto T (2012). Letter:Annular atrophic lichen planus of the lip. Dermatol Online J.

[12] Robinson CM, Oxley JD, Weir J, Eveson JW (2006). Lichenoid and granulomatous stomatitis: an entity or a non-specific inflammatory process?. J Oral Pathol Med.

[13] Sato M, Tokuda N, Fukumoto T, Mano T, Sato T, Ueyama Y (2006). Immunohistopathological study of the oral lichenoid lesions of chronic GVHD. J Oral Pathol Med.

[14] De Argila D, Gonzalo A, Pimentel J, Rovira I (1997). Isolated lichen planus of the lip successfully treated with chloroquine phosphate. Dermatology.

[15] Holmukhe S, Gutte RM, Sirur S (2012). Letter: Isolated annular lichen planus of lower lip. Dermatol Online J.

[16] Gencoglan G, İnanir İ, Sahin O, Gunduz K (2011). Imiquimod 5% cream for isolated lichen planus of the lip. J Dermatolog Treat.

[17] Georgakopoulou EA, Achtari MD (2017). Oral lichenoid lesions of the upper lip. J Dermatol Case Rep.

[18] Padilha CML, Picciani BLS, Santos BM, Silva JA, Dias EP (2014). Comparative analysis of Gram's method and PAS for the identification of Candida spp. samples from the oral mucosa. J Bras Patol Med Lab.

